# Genomic evidence of *Escherichia coli* gut population diversity translocation in leukemia patients

**DOI:** 10.1128/msphere.00530-24

**Published:** 2024-10-04

**Authors:** Julie Marin, Violaine Walewski, Thorsten Braun, Samira Dziri, Mélanie Magnan, Erick Denamur, Etienne Carbonnelle, Antoine Bridier-Nahmias

**Affiliations:** 1 Université Sorbonne Paris Nord, INSERM, IAME, Bobigny, France; 2 APHP, HUPSSD, Hôpital Avicenne, Service de Microbiologie clinique, Bobigny, France; 3 Université Paris Cité, INSERM, IAME, and APHP, Hôpital Bichat, Laboratoire de Génétique Moléculaire, Paris, France; University of Michigan-Ann Arbor, Ann Arbor, Michigan, USA

**Keywords:** *Escherichia coli*, evolution, whole-genome sequencing, infection, immunosuppression, adaptation, blood stream infection (BSI), genomics

## Abstract

*Escherichia coli,* a commensal species of the human gut, is an opportunistic pathogen that can reach extra-intestinal compartments, including the bloodstream and the bladder, among others. In non-immunosuppressed patients, purifying or neutral evolution of *E. coli* populations has been reported in the gut. Conversely, it has been suggested that when migrating to extra-intestinal compartments, *E. coli* genomes undergo diversifying selection as supported by strong evidence for adaptation. The level of genomic polymorphism and the size of the populations translocating from gut to extra-intestinal compartments is largely unknown. To gain insights into the pathophysiology of these translocations, we investigated the level of polymorphism and the evolutionary forces acting on the genomes of 77 *E. coli* isolated from various compartments in three immunosuppressed patients. Each patient had a unique strain, which was a mutator in one case. In all instances, we observed that translocation encompasses much of the genomic diversity present in the gut. The same signature of selection, whether purifying or diversifying, and as anticipated, neutral for mutator isolates, was observed in both the gut and bloodstream. Additionally, we found a limited number of non-specific mutations among compartments for non-mutator isolates. In all cases, urine isolates were dominated by neutral selection. These findings indicate that substantial proportions of populations are undergoing translocation and that they present a complex compartment-specific pattern of selection at the patient level.

**IMPORTANCE:**

It has been suggested that intra and extra-intestinal compartments differentially constrain the evolution of *E. coli* strains. Whether host particular conditions, such as immunosuppression, could affect the strain evolutionary trajectories remains understudied. We found that, in immunosuppressed patients, large fractions of *E. coli* gut populations are translocating with variable modifications of the signature of selection for commensal and pathogenic isolates according to the compartment and/or the patient. Such multiple site sampling should be performed in large cohorts of patients to gain a better understanding of *E. coli* extra-intestinal diseases.

## INTRODUCTION

Bloodstream infections (BSIs) are still a major concern among onco-hematologic patients, influenced by factors such as the type of pathogen, the degree of host immunodeficiency, and the status of underlying disease. Despite advances in the clinical management of hematological malignancies, BSIs remain life-threatening complications in the clinical course of these patients, with the reported crude mortality rate up to 40% (1–3). A clear shift in the bacterial species causing BSIs in patients with hematological malignancies has been recently reported, transitioning from Gram-positive to Gram-negative, in the first place Enterobacteriaceae, and in particular *Escherichia coli*, represent the most frequently involved bacterial species, together with the worrisome and growing phenomenon of multiresistant bacteria (4, 5).

*E. coli* is a commensal species of the lower intestine of humans (6). The gut is its primary habitat and probably the main ecological context of selection. Virulence genes, for instance, are thought to be primarily selected in the intestine as a by-product of commensalism (7, 8). *E. coli* is also an opportunistic pathogen, frequently responsible for intestinal and extra-intestinal infections (9). When reaching a new compartment, such as the bladder or the bloodstream, *E. coli* faces new challenges and new opportunities for adaptation.

Previous studies have mainly investigated the signatures of selection in commensal *E. coli* isolates and those sampled from extra-intestinal infections, revealing different scenarios. The evolution of commensal *E. coli* has been found to be governed by purifying selection, whether it implies the entire genome (10) or specific genes such as the H7 flagellin genes (11). However, another study, following the evolution of a clone in a single individual, did not find any evidence for selection in the gut (12). On the contrary, adaptation during chronic and acute infections has been observed (13). In particular, it has been shown that *E. coli* isolates colonizing extra-intestinal sites were adapting under strong selective pressure, with an excess of nonsynonymous mutations and patterns of convergence at the gene level (including for H7 flagellin genes). Evidence for adaptation during chronic infection has been emphasized for other bacteria, such as *Burkholderia dolosa* (14) or *Pseudomonas aeruginosa* (15–17), with genotypically and phenotypically diversifying lineages during cystic fibrosis infections, for instance (18–21). Adaptation to the human host (22, 23), evasion from the immune response (24, 25), and acquisition of antibiotic resistances (26, 27) are favored by this accumulation of mutations. In addition, niche adaptation shapes the allelic diversity among compartments, with specific mutations occurring in the gut or in the bladder associated with functions, increasing *E. coli* fitness in each respective compartment (28).

What happens when the intestinal barrier and the immune response are weakened? Patient deficiencies, such as immunosuppression and weakening of the intestinal barrier by antibiotic therapy or chemotherapy, can increase the risk of *E. coli* extra-intestinal infections (29–31). Anticancer chemotherapy drugs directly impact the intestinal microbiota (32), leading to dysbiosis and subsequent intestinal mucositis, which increase the risk of bacterial translocation to the bloodstream (33). Moreover, among patients undergoing chemotherapy, those with leukemia are highly prone to extra-intestinal infections and relapse (34). The weakening of intestinal barriers and the lack of immune defense could alter the adaptive conditions described for commensal and pathogenic isolates of *E. coli* in non-immunosuppressed patients. Modifications of the signature of selection are therefore expected for commensal and pathogenic isolates of *E. coli* in immunosuppressed patients. In addition, the level of genomic polymorphism and the proportion of the population translocating are largely unknown.

Here, we evaluated the selective forces acting on *E. coli* evolution in three immunosuppressed patients among three compartments: bladder, bloodstream, and gut. For each patient, we analyzed whole-genome sequences from isolates found in each compartment to assess the genetic diversity and the strength of the various selective processes at play.

## MATERIALS AND METHODS

### Sampling

Clinical isolates were isolated from three patients with leukemia and *E. coli* sepsis hospitalized in Avicenne hospital (Seine-Saint-Denis, France). Patient A experienced three infectious episodes, while patient B and C had one each. For each episode, these patients had at least one blood culture positive for *E. coli*. Isolates were sampled from positive blood culture and from bladder and feces samples on the same or subsequent day. The initial blood culture was obtained before any antibiotic therapy in patients newly admitted to the hospital. All procedures performed were in accordance with the ethical standards of the responsible committee on human experimentation (institutional and national), validated by the ethics committee of Avicenne hospital (Comité Local d’Ethique d’Avicenne). Information on the type and status of hematologic disease; presence of neutropenia (<0.5 × 10^9^/L); previous exposure to any antibiotic therapy, including prophylaxis or treatment of prior infectious episodes; type of infection; microbiological isolate; and outcome was collected in a database.

### Patient characteristics

The three patients (A to C) studied had hematological pathologies: two with acute myeloid leukemia and one with Hodgkin’s lymphoma. Patient A was diagnosed with acute myeloid leukemia and myelofibrosis in January 2014 (acute myeloblastic leukemia with minimal maturation M1), treated with an allograft. He experienced relapse in March 2015, with febrile neutropenia and presence of circulating blasts. During the BSI, the patient presented with an inflammatory syndrome and a severe immunosuppression and had received antibiotics in the previous 30 days. Patient B suffered from undifferentiated acute myeloblastic leukemia (M0), diagnosed and allografted in 2015. At the time of BSI, he was receiving chemotherapy, had severe immunosuppression, an inflammatory syndrome, and had received antibiotics within the preceding 30 days. Patient C was diagnosed with Hodgkin’s lymphoma in 2009. Relapse occurred in 2017 during the BSI episode, with the patient exhibiting inflammatory syndrome and severe immunosuppression and had previously received antibiotics.

### Prior *in vitro* phylogroup typing

The phylogroups of each sample (blood, urine, or feces) were determined using quadruplex PCR (35) to select isolates belonging to the same phylogroup for each infection episode.

### Genome sequencing and assembling

Whole-genome sequencing was performed on each sample using Illumina Technology (MiSeq and HiSeq 2500) and Nextera XT library preparation kits as instructed by the manufacturer (Illumina, San Diego, CA). Fastq files (raw sequencing data) were submitted to the European nucleotide archive (see Table S1 for accession numbers). Genome assembly was performed with SPAdes v.3.15.5 (36) (see Table S1 for assembly quality results).

For each patient, one strain was chosen as a reference and was sequenced with the Oxford Nanopore Technologies MinION platform using an R9.4 flow cell. We prepared the samples with kits LSK-108 and NBD-104 for library preparation and barcoding. The Nanopore reads were filtered with Filtlong (37) using the following parameters: minimum length of 1,000 and 95% of the best reads kept. High-quality assemblies of the three reference genomes were assembled with a hybrid strategy, using both Illumina and Nanopore reads with Unicycler V0.4.4 (38). Information on the hybrid assembly quality is presented in Table S2.

### Core and accessory genome

Seventy-seven SPAdes assemblies were annotated with Prokka (39). Plasmid sequences were predicted by PlaScope (40). Pan-genome analysis from annotated assemblies was performed with Roary using default parameters (41). The core genome alignment and the list of genes of the accessory genome were generated for the three patients.

### Variant calling (SNPs and deletions)

Bases with a low quality score (<30) were discarded, and the adapters were removed with Trim Galore (a wrapper of the Cutadapt program [42]). SNPs were detected by aligning the reads to the corresponding reference sequence of each patient (Table S1) with Snippy 4.4.0 (43) with the following parameters: the nucleotide minimum quality to be analyzed (basequal) is equal to 20, the minimum number of reads covering a site (mincov) is equal to 10, and the minimum proportion of those reads different from the reference (minfrac) is equal to 0.9. Structural variants (deletions) were detected by mapping reads to the corresponding reference assembly with BWA-MEM (44), and then sequence alignment map (sam) files were analyzed with Wham (45). As recommended, we remove calls smaller than 50 bp and larger than 2 Mb. For each strain, we also removed calls with less than five supporting reads.

### Detection of insertion sequence (IS) elements

IS elements on the three reference sequences were identified with ISFinder (46). We selected hits with an e-value lower than 10e-10, a minimum alignment coverage of 50%, and a minimum identity of 70%. Next, we searched for differences in the IS repertoire for each isolate against the corresponding reference with panISa v0.1.6 (47).

### Typing and genotypic antibiotic resistance and virulence

We used an in-house script, Petanc (48), that integrates several existing bacterial genomic tools to perform the typing of isolates with several genotyping schemes using the genomic tool SRST2 (49). Sequence types (STs) were defined using the Warwick MLST scheme and the Pasteur scheme (50, 51). We only used the Warwick scheme for the analyses described hereafter. We also determined the O:H serotypes and the *fimH* alleles (52, 53). The phylogroups were confirmed using the ClermonTyping method (54).

The resistome and virulome were first established using the in-house script Petanc (48). They were defined by BlastN with Abricate (https://github.com/tseemann/abricate) using the ResFinder (version 4.2.2) database (55), a custom database including the VirulenceFinder database and VFDB (56, 57), to which we added selected genes (58). We set the threshold for minimum identity to 80% with a minimum coverage of 90%. Next, a pan-resistome and pan-virulome were built including all the antibiotic resistance and virulence associated genes of all isolates. We mapped the reads to the corresponding pan-resistome and pan-virulome with BWA-MEM (44). We considered a gene as present when we found more than 80% coverage and at least one read. We also tested more conservative thresholds with 80% coverage and more than five reads.

To evaluate the possibility of false-negative results, we applied the same methodology for 14 MLST genes (*adk*, *dinB*, *fumC*, *gyrB*, *icd*, *mdh*, *pabB*, *polB*, *purA*, *putP*, *recA*, *trpA*, *trpB*, and *uidA*) and for genes encoded on a plasmid (predicted by PlaScope [40]), 157, 381, and 157 genes for patients A, B, and C, respectively).

### Genomic diversity and traces of selection

Rates of nonsynonymous and synonymous mutation were compared by computing nonsynonymous substitution / synonymous substitution (dN/dS) ratios (R language (59)) from the gene alignments obtained with Roary to evaluate the genomic traces of selection. To be able to compare the isolates taking into account their phylogenetic history, we reconstructed an ancestral sequence for each patient to which each isolate was then compared. We first midpoint rooted the trees of patient B and C for which there was only one sampling time. For patient A, we rooted the tree based on the best root-to-tip correlation (function “initRoot,” package BactDating [60]). Next, we inferred the ancestral sequence as the sequence at the root of each tree using parsimony (function ancestral.pars; package phangorn [61]). We calculated dN and dS as the observed number of substitutions of each type divided by the number of potential substitutions of the same type in the considered sequence. For each codon, the number of potential nonsynonymous or synonymous substitutions is determined by the genetic code. We then computed the dN/dS ratio for each pair of sequences and determined the mean dN/dS ratio and assessed whether it significantly differed from 1 (Wilcoxon test), where 1 represents perfect balance between diversifying and purifying selection, indicating no visible selection. When in a pair of sequences, one or more nonsynonymous and zero synonymous mutations are encountered, an infinite value is returned. To take into account these nonsynonymous mutations, we used the Laplace smoothing technique (62), which is used to overcome issues caused by certain values having zero occurrence. We computed the standard deviation of all possible synonymous mutations and added this value to each term of the dN/dS ratio. When there was no mutation, neither synonymous nor nonsynonymous, we removed this comparison because this case did not correspond to any of the three categories, diversifying, purifying, or neutral selection.

To examine changes that occurred specifically in each compartment, we used the same methodology and computed dN/dS ratios for clades grouping samples of the same compartment. In this study, the ancestral sequence was defined as the inferred sequence at the root of the focal clade.

## RESULTS

**Fig 1:**
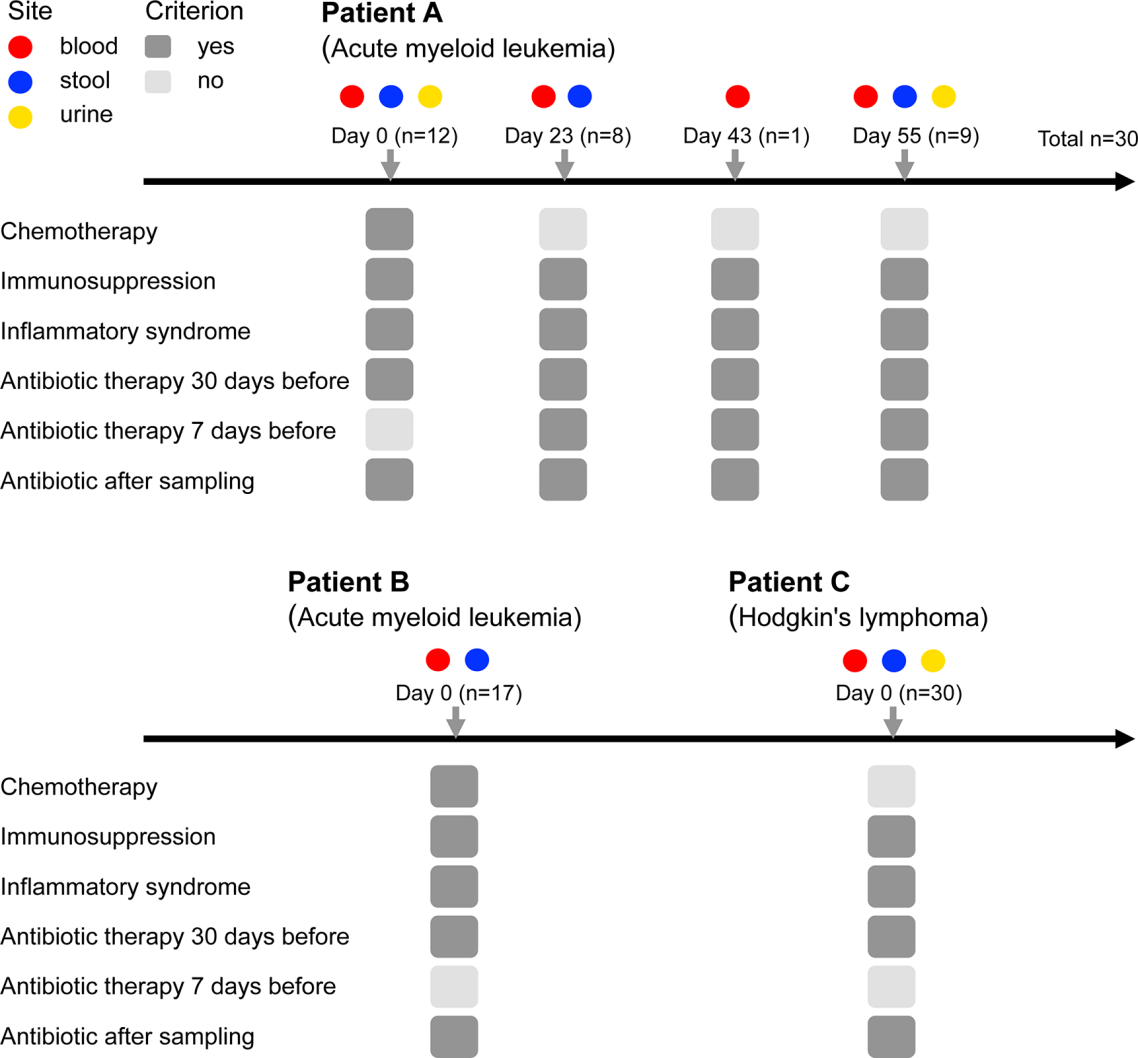
Patient follow-up with their main clinical characteristics and the sampling schemes. Absence (light gray) or presence (dark gray) of each criterion is shown. Patient A had four infectious episodes, and patients B and C had one infectious episode each.

We evaluated the genomic diversity (SNPs and deletions) and the genomic traces of selection in 77 *E. coli* isolates sampled concomitantly from the gut, blood, and urine compartments of three patients (Fig. 1). The distribution of isolates for each patient and compartment is detailed in Table 1.

**TABLE 1:** Patient sampling and strain typing

Patient	Number of isolates (stool–blood–urine)^*a*^	Phylogroup	Sequence type	Serotype	*fimH* allele
Patient A	30 (13–12–5)	A	10	O101:H9	*fimH*54
Patient B^*b*^	17 (10–7)	B1	1431	O8:H19	*fimH*32
Patient C	30 (10–10–10)	B2	131	O25b:H4	*fimH*30

^*a*^ Patients B and C were sampled at a single time point, whereas samples of patient A correspond to four time points.

^*b*^ Phylogroups, MLSTs (Warwick and Pasteur), serotypes, and *fimH* alleles are indicated. All the isolates of patient B were mutators.

### Phylogroup, ST, serotype, and *fimH* allele diversity

Within each infection episode, all isolates belonged to the same phylogroup and ST and had the same serotype and *fimH* allele (Table 1). Isolates belonged to the phylogroup A (patient A), B1 (patient B), and B2 (patient C).

### Global genomic diversity (SNPs and IS)

We first looked at the SNP diversity. With the exception of patient B, isolates exhibited a low number of mutated genes (between eight and 11) and no deletion (Table S3). Patient B isolates, however, revealed 328 mutated genes. Indeed, we noted a deletion of *mutS* in patient B isolates. This inactivation of the DNA mismatch repair system conferred to them a mutator phenotype (63, 64) (Table S4).

**Fig 2:**
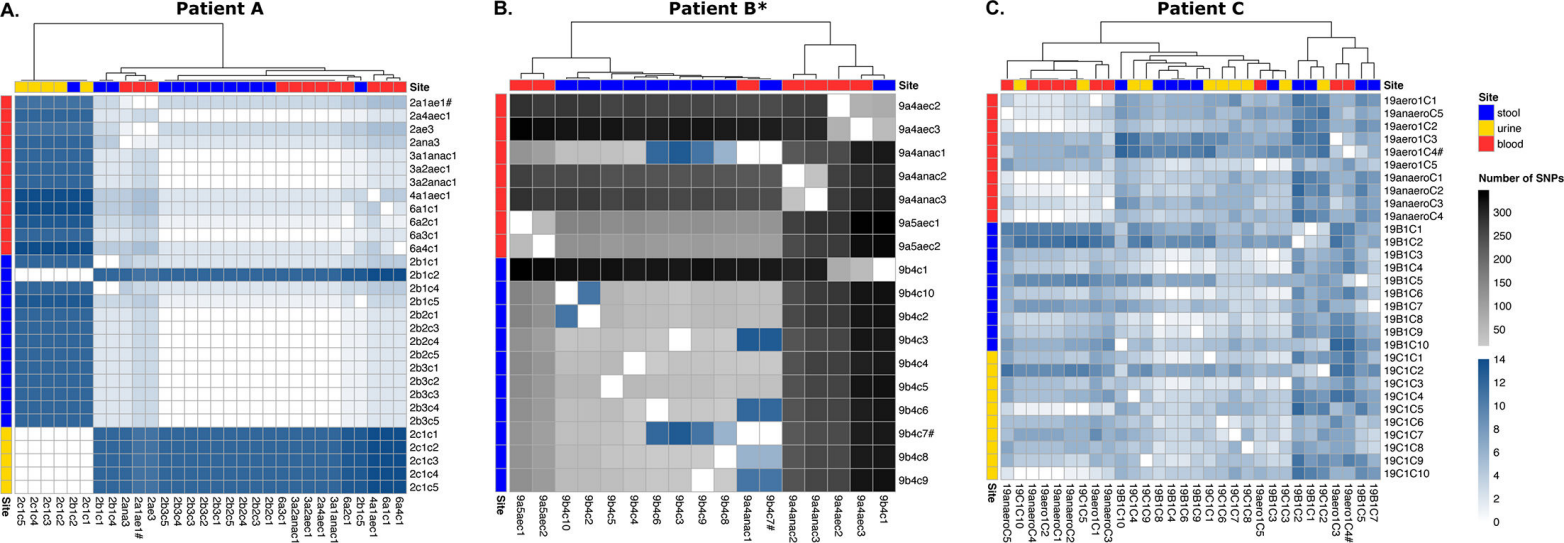
Genomic diversity of *E. coli* isolates among samples and compartments for the three patients. (**A–C**) Heatmaps showing the number of SNPs between each isolate of patients (**A–C**). Samples are ordered by site horizontally and clustered according to their SNP similarities vertically (method: complete). All isolates of patient B are mutators (*). The isolate corresponding to the reference sequence is indicated (#). We used the same color scale for all patients. Note that the number of SNPs for patients A and C is between 0 and 14 and between 0 and 400 for patient B.

In line with those results, we found a low number of SNPs in isolates from patients A and C (mean number of 5.07 [95% CI: 4.20–5.95]) compared to those of patient B (mean number of 117.13 [82.16–152.10], *P*-value = 1.237e-08 (Wilcoxon test)) (Fig. 2). Similarly, intra- and extra-intestinal isolates did not differ significantly in term of variant categories (Table 2) (chi-squared test after correcting for sample size, *P*-value = 0.99). No common genes with SNPs (Table S5) were identified among patients (Table S3).

**TABLE 2:** Number of DNA variant types in coding regions among isolates of a patient’s infection episode found with Snippy^*a*^

	Site	Conservative inframe insertion	Disruptive inframe deletion	Frameshift variant	Missense variant	Nonsense variant	Stop codon lost variant	Synonymous variant	tRNA variant
Patient A	Blood (*n* = 12)	9	0	1	3	1	0	9	0
	Stool (*n* = 13)	12	1	0	6	0	1	17	0
	Urine (*n* = 5)	0	5	0	20	0	5	20	0
Patient B^*b*^	Blood (*n* = 7)	0	0	216	541	11	20	292	5
	Stool (*n* = 10)	0	0	86	177	4	4	146	1
Patient C	Blood (*n* = 10)	0	0	9	7	0	0	2	0
	Stool (*n* = 10)	0	0	16	26	0	0	14	0
	Urine (*n* = 10)	0	0	12	20	0	0	6	0
Intra-intestinal isolates	Stool (*n* = 23)	12	1	16	32	0	1	31	0
Extra-intestinal isolates	Blood/urine (*n* = 37)	9	5	22	50	1	5	37	0

^*a*^ *n*: number of isolates.

^*b*^ We excluded patient B from intra- and extra-intestinal isolates because all the 17 isolates were mutators.

Additionally, the diversity in IS elements revealed only few differences among isolates of the same patient (zero to five for non-mutator strains) (Tables S6 and S7). The number of IS within the isolates of each patient was in the upper part of the range found for *E. coli* genomes (65), with a lower number of IS elements for the mutator strain, as expected (66). We detected five supplementary potential IS elements in one to 14 isolates (4.6 isolates in average) for patient A compared to the reference genome (189 IS elements). For patient B (mutator strain), we detected 22 supplementary potential IS elements in one to 11 isolates (2.63 isolates in average) compared to the reference genome (97 IS elements). We did not find any supplementary potential IS element in patient C isolates compared to the reference genome (136 IS elements).

### Within-patient compartment diversity

**Fig 3:**
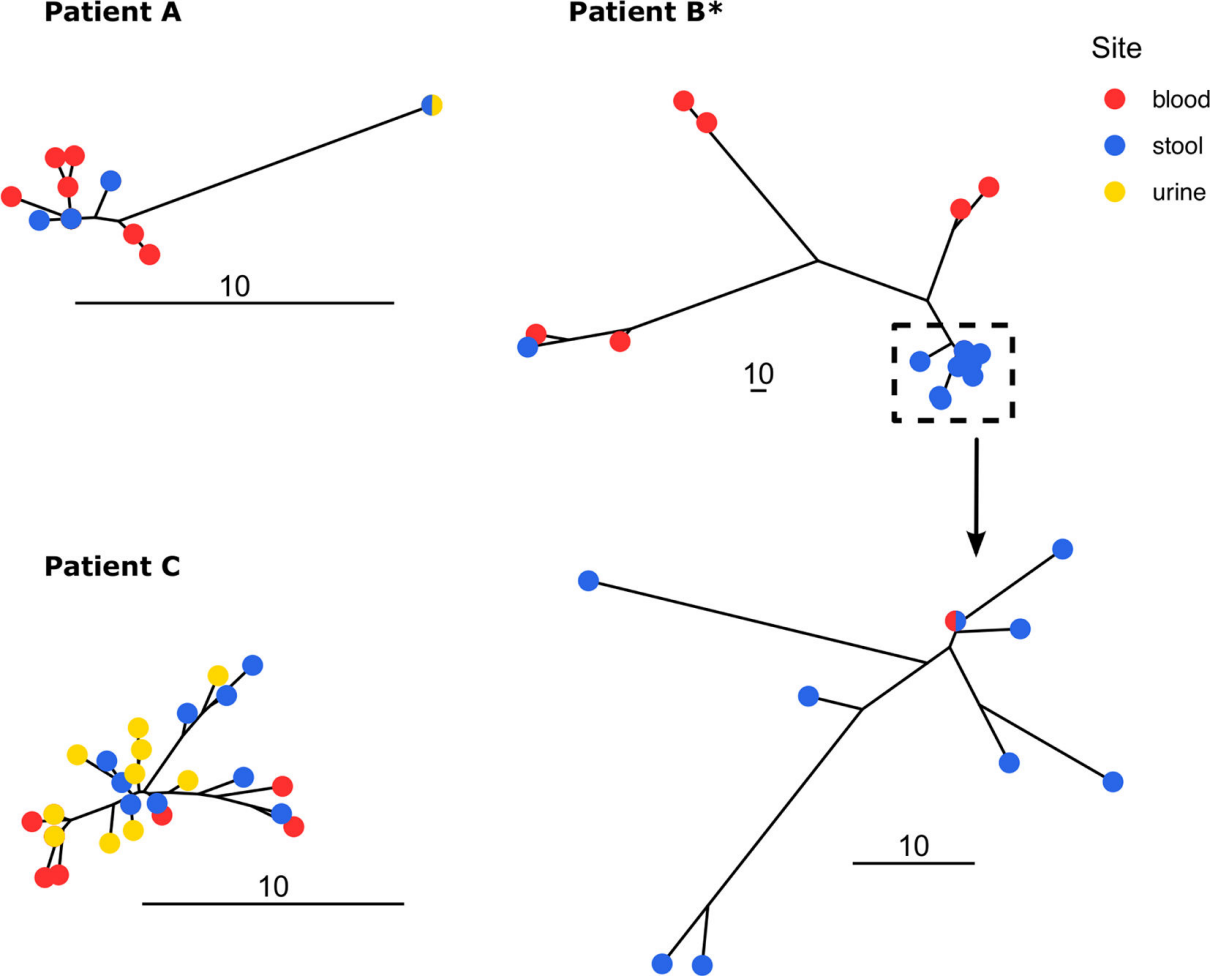
Unrooted trees of *E. coli* isolates from patients A, B, and C. Trees were built using neighbor joining from the substitution presence/absence matrix. The scale indicates the number of substitutions. All isolates of patient B are mutators (*). We zoomed in on a clade of the patient B to highlight the scale difference. The bicolor points (patients A and C) denote the presence of isolates sampled in different sites with identical sequences (zero SNP).

All isolates from a given patient were highly similar (except for patient B with mutator isolates) (Fig. 2 and 3). Consequently, no large deletions were detected because we used one of these isolates as the reference strain for each patient (Table S3).

**Fig 4:**
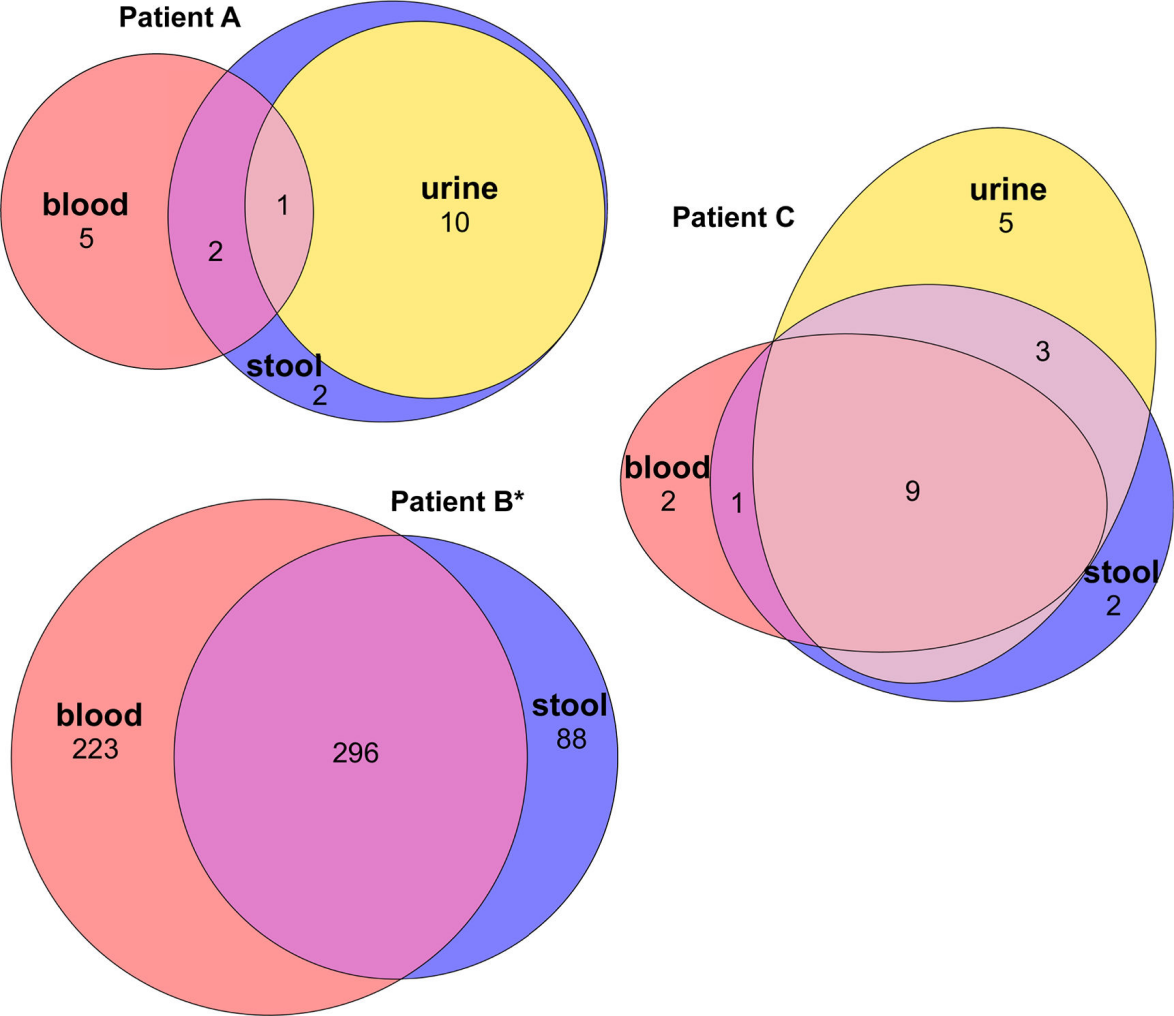
Venn diagrams showing the SNP distribution among compartments for patients A, B, and C. The ellipses are proportional to the number of SNPs. All isolates of patient B are mutators (*).

The majority of SNPs were shared among compartments (Fig. 4). For patient A, 13 over 20 SNPs were identical among at least two compartments and 13 over 22 for patient C. For patient B (mutator strain), despite a high level of polymorphism, most SNPs were shared among stool and blood isolates. Furthermore, most of the stool polymorphism was found in other compartments, 87%, 77%, and 87% for patients A, B, and C, respectively, suggesting a large population translocation from the gut.

Additional IS elements were also shared among compartments (Table S7). For patient A, three over five additional IS elements were shared among two compartments and 12 over 22 for patient B.

### Antibiotic resistance and virulence-associated gene content

**Fig 5:**
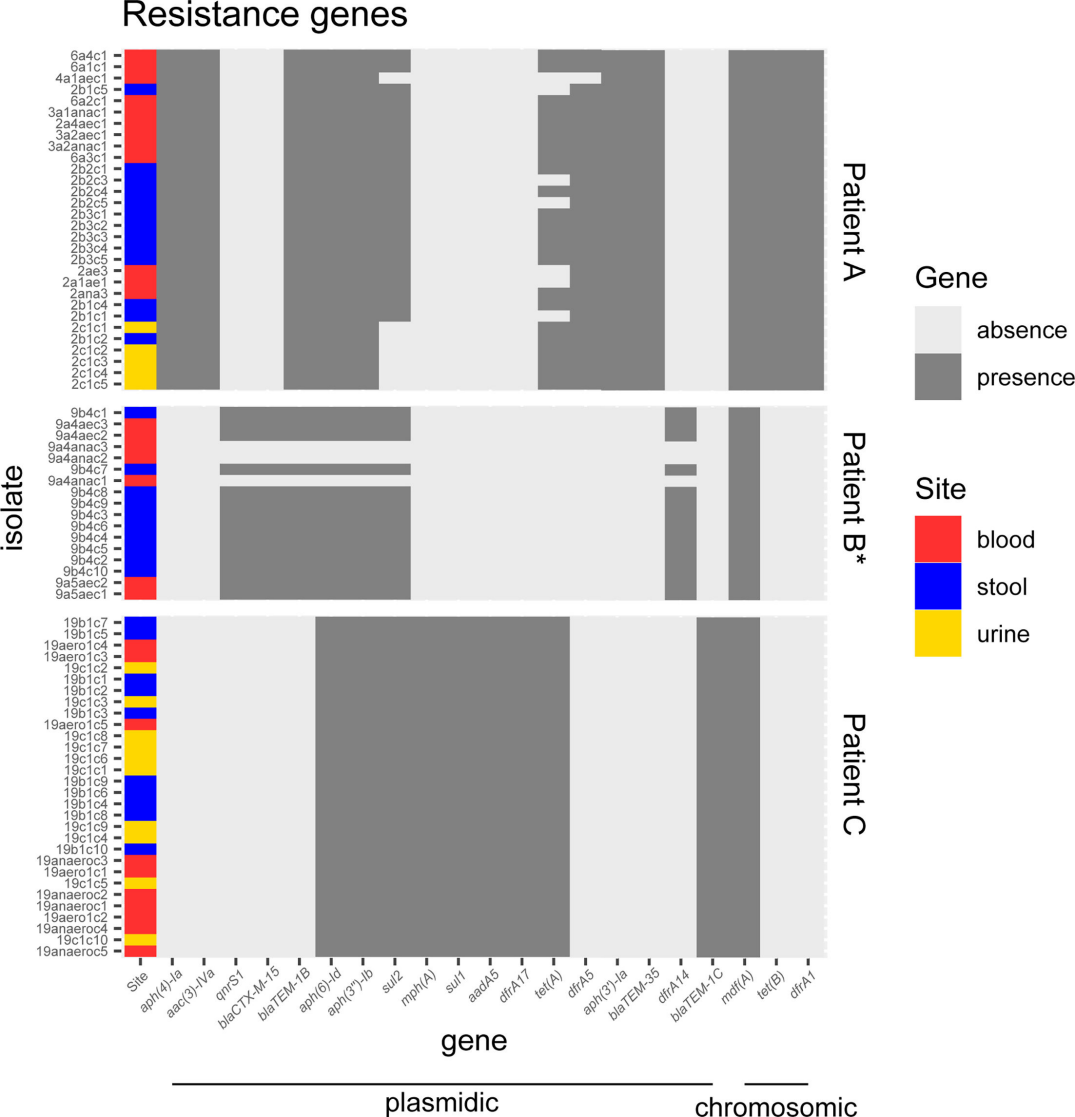
Presence/absence heatmaps of antibiotic resistance genes of *E. coli* isolates when compared to the pan-resistome (including the resistance genes of all isolates). We considered a gene as present when at least 80% of its length was covered by at least one read. Genes are ordered by synteny on contigs. All isolates of patient B are mutators (*). The prevailing predicted localization of genes by PlaScope (chromosomic or plasmidic) is indicated (full list in Table S8). Note that chromosomal genes are not mobile.

Regarding genes associated with antibiotic resistance, isolates within each infection were highly similar. Limited variations were observed among isolates of patient B (mutator isolates) and A, which were sampled at different time points (Fig. 1 and 5). For patient A, we found gene presence/absence discrepancies for three resistance genes, *sul2*, *tet*(*A*), and *dfrA5*. The gene *sul2* was consistently predicted as encoded on a plasmid across all isolates (Table S8). The genes *dfrA5* and *tet*(*A*) were found on the same contig in 20 isolates out of 23 isolates possessing both genes and were predicted as encoded on a plasmid for 96.67% and 86.96% of the isolates, respectively. For patient B, we found presence/absence discrepancies for all the seven resistance genes detected. The genes *qnrS1*, *bla*CTX-M-15, *bla*TEM-1B, *aph*(6)-Id, *aph*(3′′)-Ib, and *sul2* were always co-located on the same contig and predicted as encoded on a plasmid. The gene *dfrA*14 was predicted as encoded on a plasmid for all isolates. Similar results were obtained using a more conservative threshold (at least five reads covering more than 80% of the gene) (Fig. S1), with the exception of the gene *mdf*(*A*), predicted as chromosomal, which was missing in three isolates of patient B.

**Fig 6:**
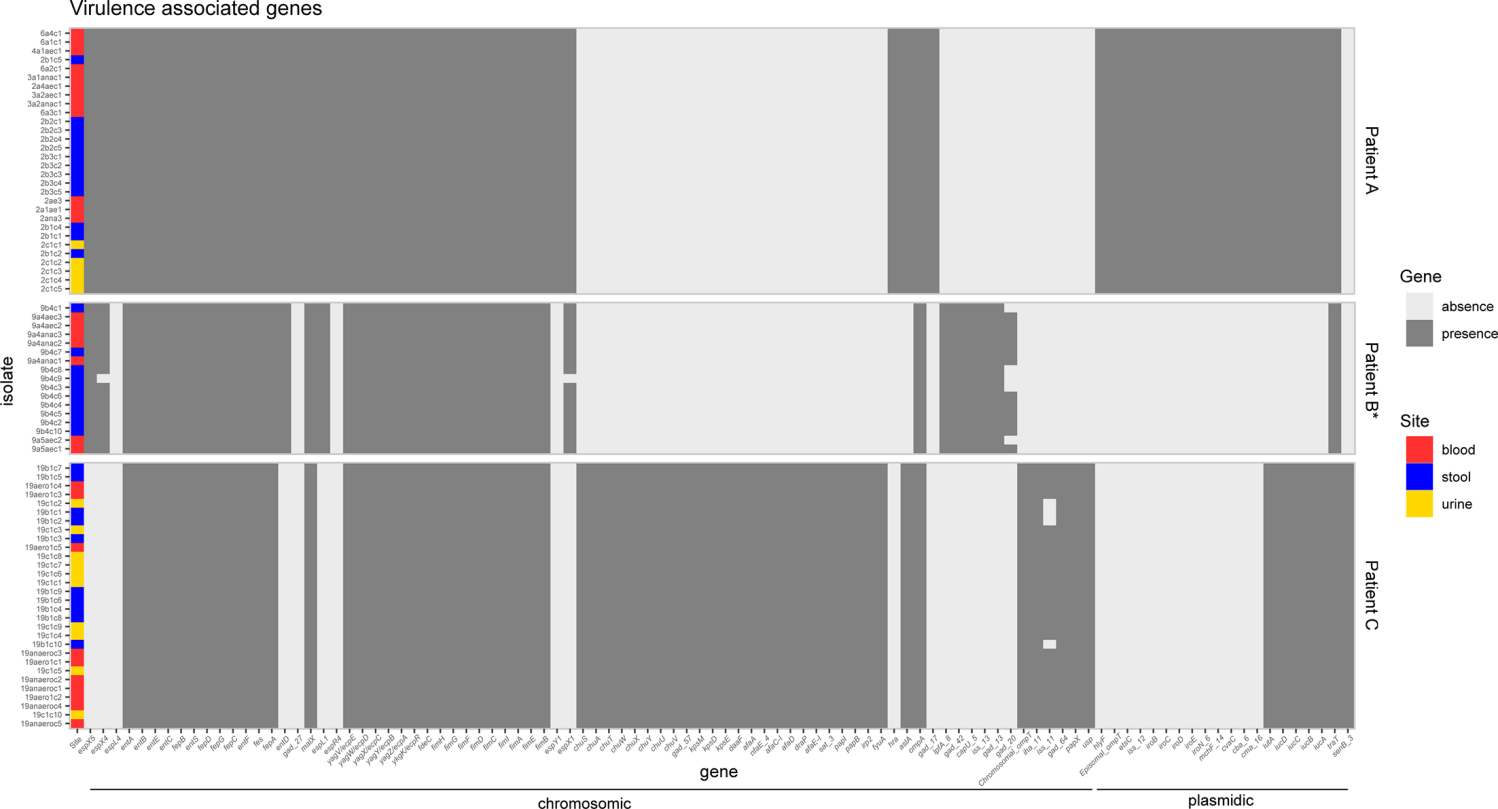
Presence/absence heatmaps of virulence-associated genes of *E. coli* isolates when compared to the pan-virulome (including the virulence genes of all isolates). We considered a gene as present when at least 80% of its length was covered by at least one read. Genes are ordered by synteny on contigs. All isolates of patient B are mutators (*). The prevailing predicted localization of genes by PlaScope (chromosomic or plasmidic) is indicated (full list in Table S9). Note that plasmidic genes are not mobile, at the opposite of resistance genes (see Fig. 5).

High gene content similarity was also observed for virulence-associated genes (Fig. 6). An identical gene content was found in patient A isolates. For patient C, we noted a discrepancy for a single gene, *iss*11, consistently predicted as chromosomal, and absent in four isolates. Slightly more differences were found in patient B isolates (mutator strain). The genes *espX5* and *espX1* were missing for one isolate and *gad*20 for five isolates, all predicted as chromosomal (Table S9). We found more discrepancies with a more conservative threshold (at least five reads covering more than 80% of the gene), highlighting lower sequencing depth of two isolates in particular (Fig. S2). In patient A, all the isolates had the same gene content, with the exception of two isolates (2ana3 and 9b4c8). We found six missing genes for 2ana3 and one missing gene for 2c1c5, all predicted as chromosomal. In addition, *entA* and *entB* genes were always adjacent on the same contig (Table S9). In patient C isolates, the gene *iss*11, always predicted as chromosomal, was missing in five isolates. Patient B displayed a greater number of differences, over the 47 virulence-associated genes detected, all predicted as chromosomal, and only 15 were present in all isolates.

As a control for false-negative results, we evaluated the presence of 14 MLST genes and of genes encoded on plasmids. We recovered all the MLST genes for all patients (Fig. S3). With a more conservative threshold (at least five reads covering more than 80% of the gene), we also found all the MLST genes for patients A and C. For patient B (mutator strain), six genes were not recovered over the 238 possibilities (Fig. S3). Regarding genes encoded on a plasmid, there were very few differences depending on the depth threshold used (Fig. S5). With a more stringent threshold, we found six additional missing genes, over 4,710 possibilities, for patient A and seven additional missing genes, over 6,477 possibilities, for patient B. We did not find any differences for patient C. For patients A and B, in most of the cases, missing genes corresponded to the absence of all the genes encoded on the corresponding plasmid. There were few exceptions that could be explained by a recent loss of the plasmid either *in vivo*, when isolates of the same cluster lack the same plasmid (e.g., plasmid 2 of patient A) or *in vitro* during the lab subcultures as we detected traces of the lost plasmids (i.e., portions of some of the genes encoded on the plasmid), suggesting the presence of the plasmid at a low frequency in the sequenced colony.

### Genomic traces of selection

**Fig 7:**
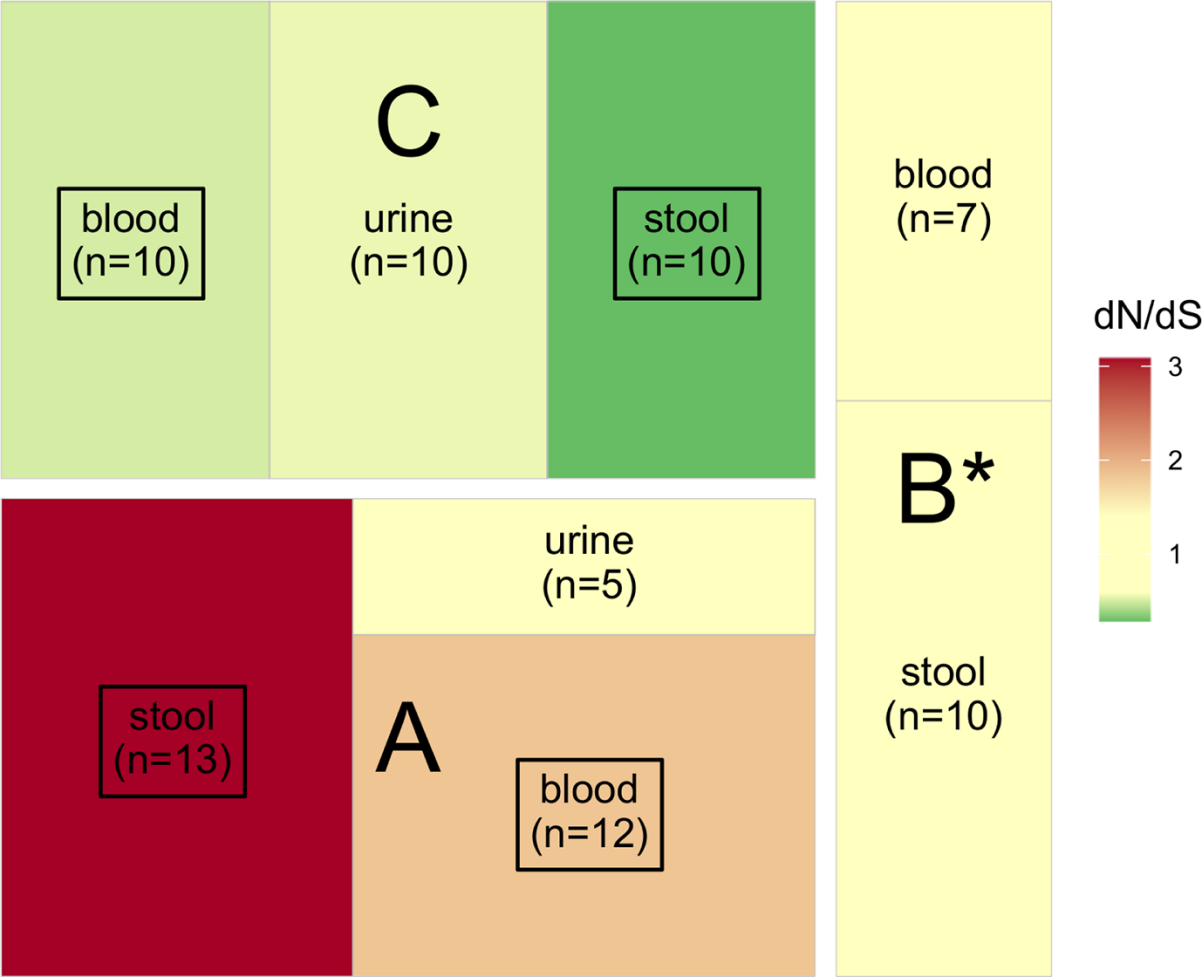
Action of selection on sequences (dN/dS) of *E. coli* isolates for patients A, B, and C. Significant results are framed in black (see Table S9). All isolates of patient B are mutators (*). Neutrality (dN/dS not significantly different from 0) is indicated in pale yellow, whereas purifying and diversifying selection are indicated in green and red, respectively.

We computed dN/dS ratios to assess whether gene sequences evolved neutrally or were under purifying or diversifying selection (Fig. 7; Table S10). As expected, all isolates of patient B were under neutral selection (67, 68). For patients A and C, the same pattern of selection was found for blood and stool isolates, diversifying selection for patient A and purifying selection for patient C. We also found that all isolates sampled in urine evolved under neutral selection (patients A and C). As such, results might be biased when the phylogenetic history is likely to include changes that occurred in other compartments, and we computed dN/dS ratios in clades grouping samples of the same compartment (stool or blood). Despite the limited number of samples preventing us from obtaining significant results, we globally found the same trends in selection patterns (Table S11).

This analysis does not compute the variation in noncoding regions; however, it encompasses more than 80% of all the variants and more than 87% of the genome. It should be noted that the number of variants (missense and synonymous) cannot be directly compared to dN/dS ratios (Table 1). These variants were computed with reference to a single sequence, whereas ratios were computed against a reconstructed ancestral sequence.

## DISCUSSION

The bacterial species *E. coli* is an opportunistic pathogen (6, 9) that may cross the intestinal barrier to reach extra-intestinal compartments, causing infections. In this context, it has been proposed that virulence could be a by-product of commensalism (8). While evidence for purifying and neutral selection has been detected in commensal *E. coli* (10–12), extra-intestinal infection isolates have been shown to be under strong adaptive selection (13). In this study, we investigated both commensal and extra-intestinal isolates from leukemia patients undergoing chemotherapy. We observed the same strain in all compartments. Isolates of patients A and C were characterized by a very limited number of SNPs, five in average (ranging from zero to 14) (69), and patient B was infected by a mutator strain (64). Interestingly, while each patient had a different selection signature, it was identical in both the bloodstream and gut. In urine, in all cases, neutral selection was at play.

### Gut origin of urine and blood immunosuppressed patient isolates

For each patient, the isolates collected from the gut, urine, and blood displayed highly similar sequences. They share the same phylogroup, ST, serotype, and *fimH* allele (Table 1), indicating that the strain most likely originated from the gut, as previously shown (31, 34). Moreover, with the exception of the mutator isolates, there were fewer than 15 SNPs between pairs of isolates of the same patient (Fig. 2), the resistance and virulence profiles were stable, and there were few differences in the IS repertoire (Fig. 5 and 6). The phylogenetic distribution of isolates, together with the SNPs and IS distribution among compartments, suggested the translocation of the gut population diversity to extra-intestinal compartments for all patients (Fig. 3 and 4).

### Same adaptation forces at play in the gut and bloodstream of immunosuppressed patients

In non-immunosuppressed patients, purifying (or neutral) evolution of *E. coli* populations has been reported in the gut (12), but not in extra-intestinal compartments, where bacterial DNA sequences are under diversifying selection with strong evidence for gene level convergence (13).

Here, we found various signatures of selection in gut isolates. However, the same adaptive process was at play in the bloodstream and in the gut for each patient. For patient B, the presence of mutators at high frequency in the population (all isolates) could be an indirect evidence for ongoing selection (70, 71). Indeed, even if they will ultimately be counter-selected (64), mutator alleles can reach 100% frequency in a population because they are associated with beneficial mutations (genetic hitchhiking) (72). In natural populations, mutators are present up to 15% frequency (73–76). Despite the low number of patients, we noted a higher frequency (33%), which might be explained by patient treatment. Indeed, some anticancer chemotherapy drugs enhance the bacterial mutagenesis, thus promoting the emergence of a mutator clone (77). The presence of mutators at high frequency could suggest a recent adaptative episode with a temporary indirect selection of this phenotype. Then, the mutator phenotype, even if the high mutation load observed might already have reduced the strain fitness, did not inhibit this *E. coli* population from causing a bloodstream infection. An alternative hypothesis would be the acquisition of a mutator clone by the patient. In this scenario, the selection of a mutator phenotype took place in another host or in the environment. Then, this bacterial population managed to colonize the gut and reached the bloodstream of patient B without being counter-selected. In patient A, diversifying selection is at play, whereas purifying selection shaped the isolates of patient C. Differences in the microbiota perturbation of these patients undergoing a specific antibiotic therapy and chemotherapy could explain this discrepancy.

Unlike in extra-intestinal infection of non-immunosuppressed patients (13), no convergence among patients was detected. Moreover, isolates were almost identical with a very low number of SNPs differentiating them (five on average) unspecific to the compartment, whereas, in non-immunosuppressed patients, niche adaptation was evidenced by specific mutations associated with isolates from the gut or the bladder (28). A weakened immune system and a permeable intestinal barrier, due to chemotherapy and antibiotic treatments, likely contribute to this this phenomenon.

### Neutral selection in the bladder of immunosuppressed patient

For both patients with urine samples, isolates evolved neutrally. Fluctuating environment or a small population size could explain the weak strength of selection observed in the commensal habitat of *E. coli*. Indeed, bacteria in the bladder face continuous adaptive challenges, including fluctuations in exposure to the host immune system, antibiotic treatments, nutrient availability, and a diverse microbial community due to an alternation between storage and voiding phases (78).

Our work obviously presents a major limitation as we evaluated few patients, which prevents us from overgeneralizing our conclusions. Collecting stool before antibiotic treatment in a severe disease is indeed very difficult to achieve in clinical practices. Nevertheless, the evaluation of larger cohorts is necessary to associate evolution patterns and the environment (patient follow-up and patient-dependent factors) and to decipher the factors linked to the selection patterns observed here.

### Conclusion

We evaluated the diversity of *E. coli* isolates in stool, blood, and urine of immunocompromised patients and found the same strain across compartments. We showed that all the diversity of the population is translocating. However, contrary to non-immunocompromised patients, we did not detect any modifications in the adaptive constraints between the gut and the bloodstream. Such multi-site sampling studies should be performed in non-immunosuppressed patients to strengthen our findings.

## Data Availability

The data generated in this study have been submitted to the NCBI BioProject database under the accession number PRJEB69525.

## SUPPLEMENTAL MATERIAL

Supplemental Figures msphere.00530-24-s0001.pdf Figures S1-S5.

Supplemental Tables msphere.00530-24-s0002.xlsx Tables S1-S11.

ASM does not own the copyrights to Supplemental Material that may be linked to, or accessed through, an article. The authors have granted ASM a non-exclusive, world-wide license to publish the Supplemental Material files. Please contact the corresponding author directly for reuse.
