# Supplementary material for: Genomic evidence of Escherichia coli gut population diversity translocation in leukemia patients
Source: mSphere. 2024 Oct 4;9(10):e00530-24. doi: 10.1128/msphere.00530-24 (PMC11520291; doi:10.1128/msphere.00530-24)
Supplement: Supplemental Figures — Figures S1-S5. [file msphere.00530-24-s0001.pdf]

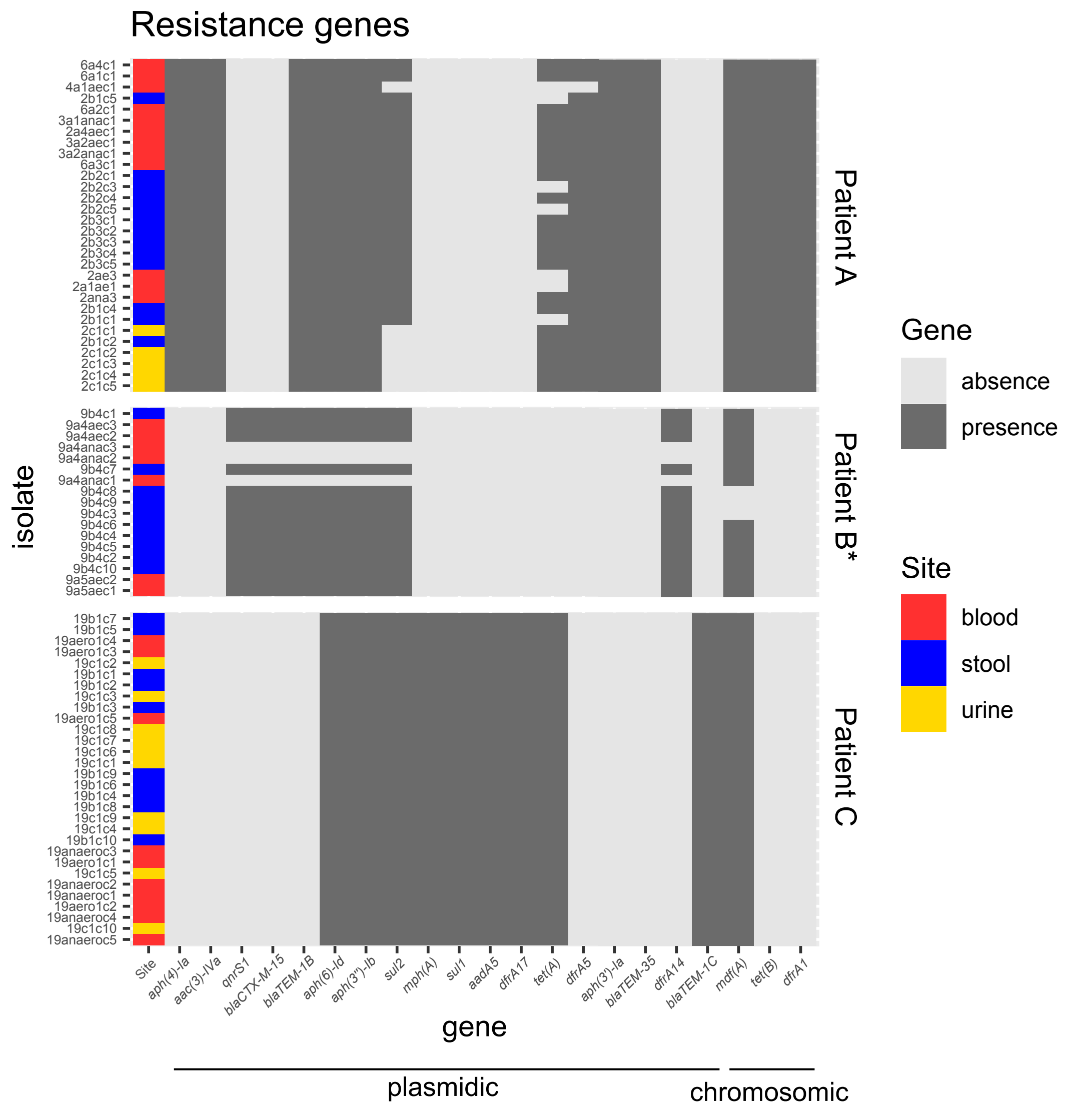

Figure S1. Presence/absence heatmaps of antibiotic resistance genes when compared to the pan-resistome (including the resistance genes of all isolates). We considered a gene as present when at least 80% of its length was covered by more than 5 reads. Genes are ordered by synteny on contigs. All isolates of patient B are mutators (*). The prevailing predicted localization of genes by PlaScope (chromosomic or plasmidic) is indicated (full list in supplementary Table S5).

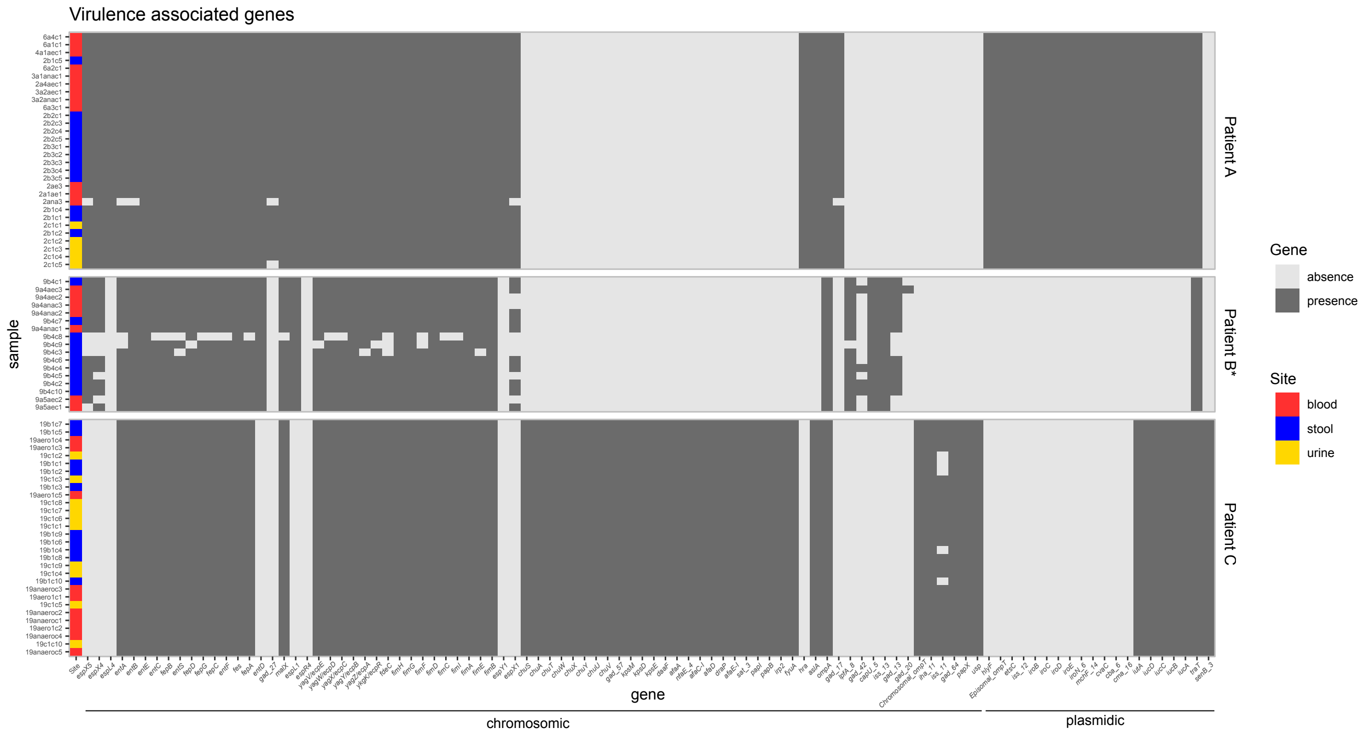

Figure S2. Presence/absence heatmaps of virulence associated genes when compared to the pan-virulome (including the resistance genes of all isolates). We considered a gene as present when at least 80% of its length was covered by more than 5 reads. Genes are ordered by synteny on contigs. All isolates of patient B are mutators (*). The prevailing predicted localization of genes by PlaScope (chromosomic or plasmidic) is indicated (full list in supplementary Table S6).

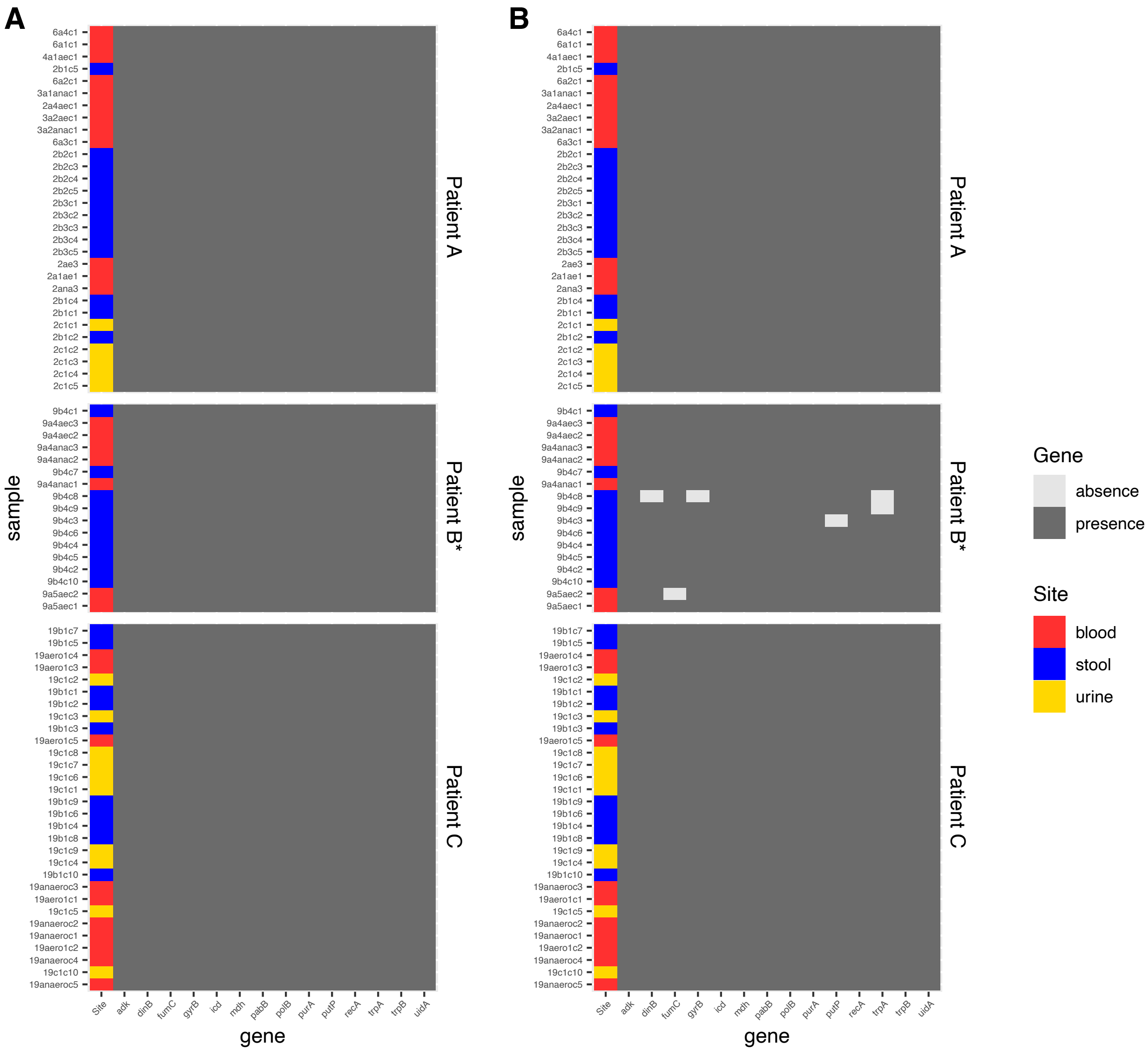

Figure S3. Presence/absence heatmaps of mlst genes. We considered a gene as present when at least 80% of its length was covered by more than 1 read (A) or by more than 5 reads (B). All isolates of patient B are mutators (*).

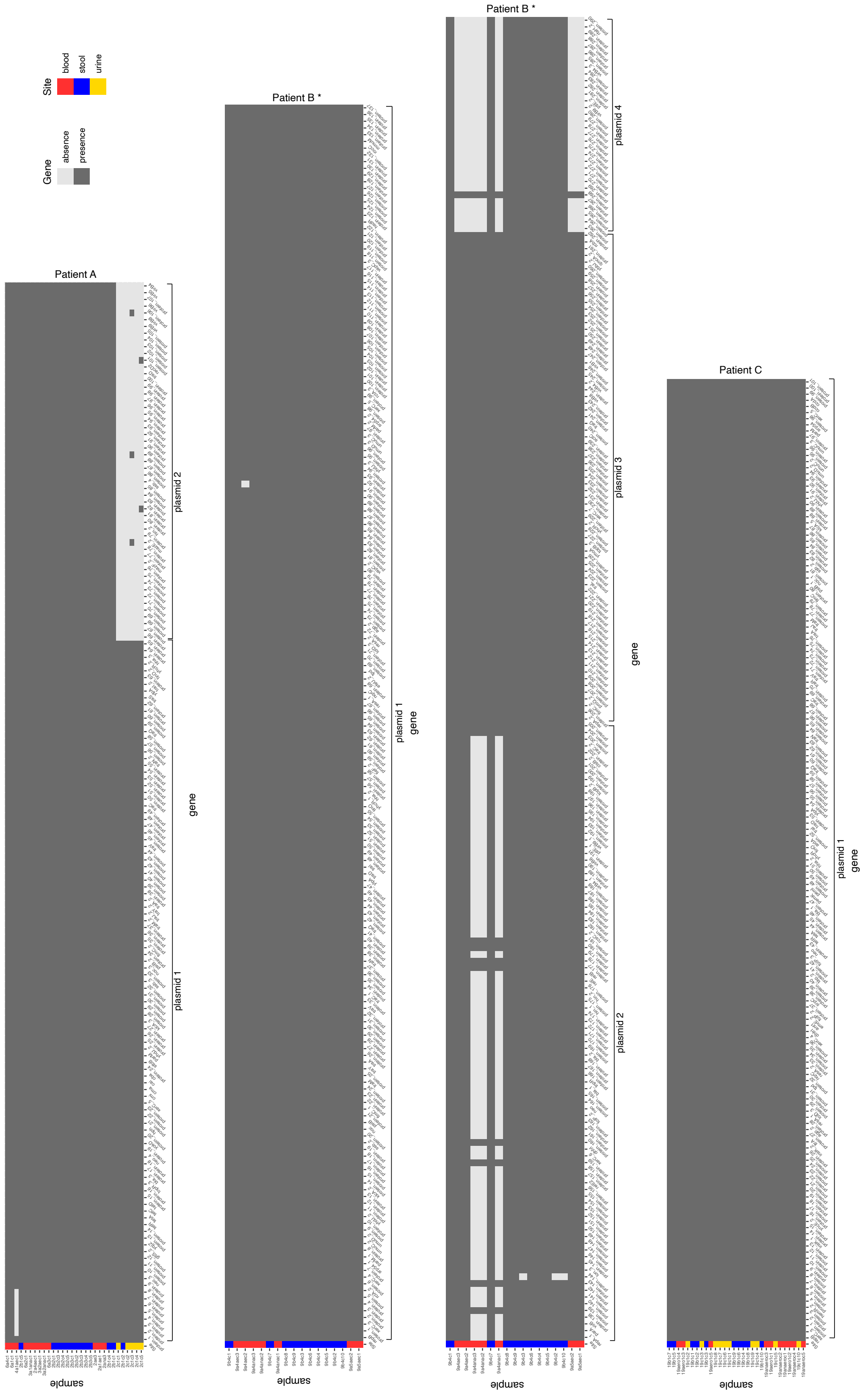

Figure S4. Presence/absence heatmaps of plasmid encoded genes. We considered a gene as present when at least 80% of its length was covered by more than 1 read. All isolates of patient B are mutators (*). Genes are ordered by synteny on contigs.

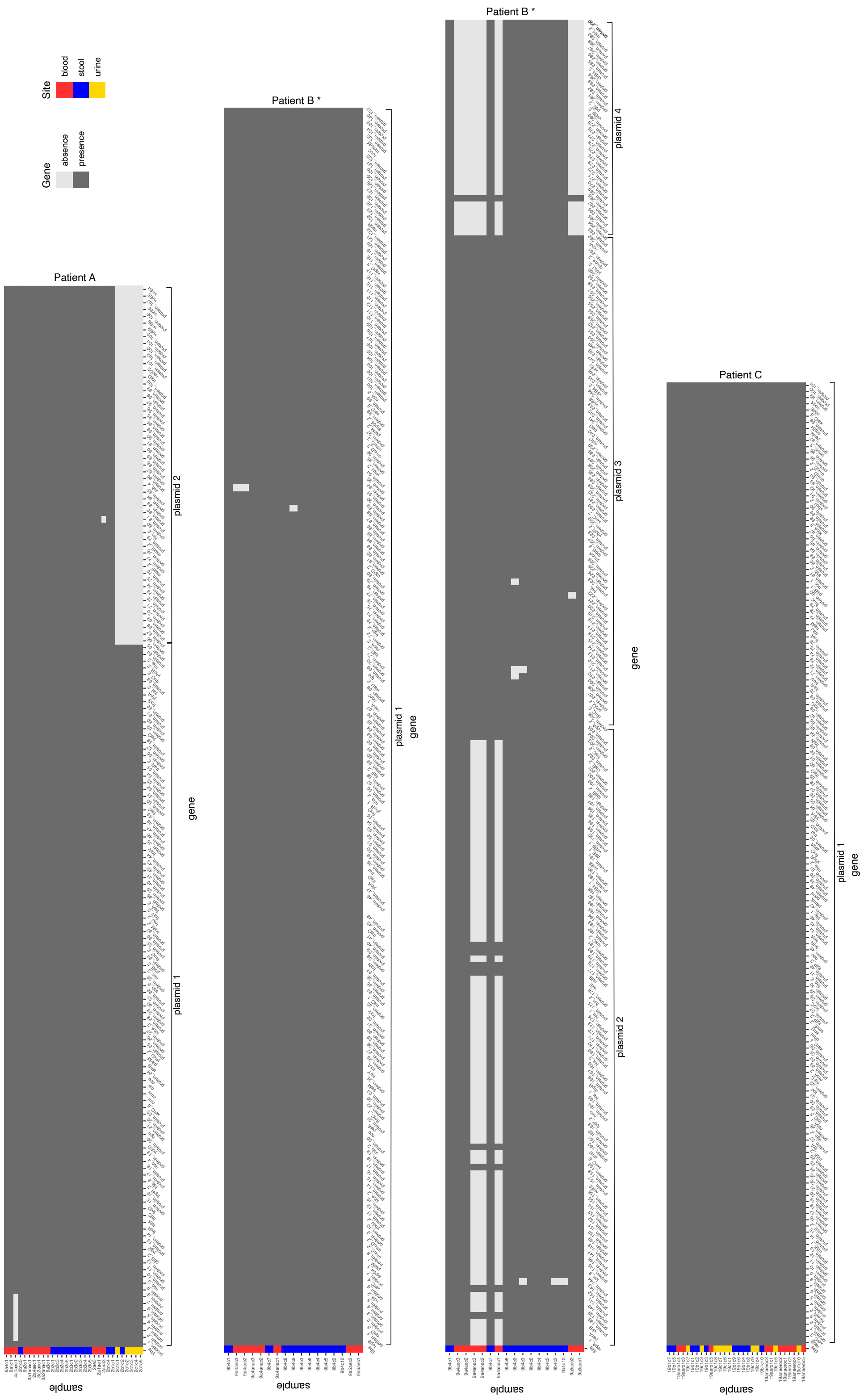

Figure S5. Presence/absence heatmaps of plasmid encoded genes. We considered a gene as present when at least 80% of its length was covered by more than 5 reads. All isolates of patient B are mutators (*). Genes are ordered by synteny on contigs.
